# Serum cytokine responses in *Rickettsia felis* infected febrile children, Ghana

**DOI:** 10.1007/s00430-018-0544-3

**Published:** 2018-05-08

**Authors:** Jessica Rauch, Peter Sothmann, Cassandra Aldrich, Ben Hogan, Ellis Owusu-Dabo, Jürgen May, Daniel Eibach, Dennis Tappe

**Affiliations:** 1 Bernhard Nocht Institute for Tropical Medicine, Bernhard-Nocht-Str. 74, 20359 Hamburg, Germany; 2 Division of Tropical Medicine, 1st Department of Medicine, University Medical Center Hamburg Eppendorf, Hamburg, Germany; 3 German Center for Infection Research (DZIF), Partner Site Hamburg-Borstel-Lübeck, Brunswick, Germany; 4 Kumasi Centre for Collaborative Research in Tropical Medicine, Kwame Nkrumah University of Science and Technology, Kumasi, Ghana; 5 School of Public Health, KNUST, Kumasi, Ghana

**Keywords:** *Rickettsia felis*, Rickettsiosis, Flea-borne spotted fever, Cytokine

## Abstract

The intracellular pathogen *Rickettsia felis* causes flea-borne spotted fever and is increasingly recognized as an emerging cause of febrile illness in Africa, where co-infection with *Plasmodium falciparum* is common. Rickettsiae invade endothelial cells. Little is known, however, about the early immune responses to infection. In this study, we characterize for the first time the cytokine profile in the acute phase of illness caused by *R. felis* infection, as well as in plasmodial co-infection, using serum from 23 febrile children < 15 years of age and 20 age-matched healthy controls from Ghana. Levels of IL-8 (interleukin-8), IP-10 (interferon-γ-induced protein-10), MCP-1 (monocyte chemotactic protein-1), MIP-1α (macrophage inflammatory protein-1α) and VEGF (vascular endothelial growth factor) were significantly elevated in *R. felis* mono-infection; however, IL-8 and VEGF elevation was not observed in plasmodial co-infections. These results have important implications in understanding the early immune responses to *R. felis* and suggest a complex interplay in co-infections.

## Introduction

*Rickettsia felis* causes flea-borne spotted fever (FBSF), an acute febrile illness commonly involving headache, myalgia and rash and potentially leading to severe neurological and respiratory complications [1]. While distributed worldwide, *R. felis* infection is increasingly reported in Africa, where prevalence rates of 3–15% in acute fever episodes suggest an emerging importance as a cause of febrile illness [2–5]. The obligate intracellular pathogen *R. felis* belongs to the transitional group of rickettsiae, as it shares phenotypic characteristics with members of the spotted fever group (SFG) and the typhus group (TG) [2]. Rickettsiae cause endothelial cell (EC) infection which can lead to vasculitis and bacterial dissemination [6]. The vascular permeability observed in clinical cases seems to be mediated at least in part by inflammatory cells and their mediators [7]. ECs, that are besides macrophages the major target cells for rickettsial infections [8–12], react to infection with TG and SFG rickettsia with the production of proinflammatory cytokines like IL-1 (interleukin-1), IL-6 and TNFα (tumor necrosis factor-α), chemokines like IL-8, IP-10 (interferon-γ induced protein-10) and MCP-1 (monocyte chemotactic protein-1) and other mediators in vitro that lead to activation and recruitment of immune cells to the site of infection [8, 13–17]. IL-8, for example, promotes the recruitment of neutrophils to the site of infection and mediates angiogenesis [18–20]. IP-10 and MCP-1 are involved in the recruitment of monocytes and activated NK cells and T cells which further lead to potentiation of the inflammatory response to rickettsial infection and its clearance [21–23]. Mouse models of rickettsial infections further help to understand the role of cytokines in vivo. IFNγ (interferon-γ) and TNFα have protective properties during rickettsial infection of susceptible mouse strains [24–29]. IFNγ and TNFα activate intracellular bactericidal mechanisms; it was shown that IFNγ inhibits the growth of rickettsia in various host cells [28–33]. However, in humans, the early host immune responses have not been well-characterized. In this study, we investigated serum cytokine responses in febrile children from Ghana with acute *R. felis* infection.

## Patients and methods

Serum cytokines and chemokines were analyzed by bead-based LEGENDplex assay (BioLegend, London) from 23 febrile children < 15 years of age (age range 0–7 years, median: 2 years) with molecularly confirmed *R. felis* infection seen at St. Michael’s Hospital, Pramso, Ghana [3]. The detection limits of the LEGENDplex assay for the analyzed cytokines were as follows: bFGF (basic fibroblast growth factor: 5.03 pg/mL), G-CSF (granulocyte colony stimulating factor: 8.77 pg/mL), GM-CSF (granulocyte–macrophage colony stimulating factor: 9.44 pg/mL), IFNγ (3.08 pg/mL), IL-1ß (N/A), IL-2 (3.34 pg/mL), IL-4 (4.46 pg/mL), IL-5 (3.61 pg/mL), IL-6 (2.86 pg/mL), IL-8 (5.13 pg/mL), IL-9 (1.27 pg/mL), IL-10 (2.97 pg/mL), IL-12p70 (30.33 pg/mL), IL-13 (N/A), IL-17A (4.29 pg/mL), IL-17F (4.24 pg/mL), IL-21 (1.37 pg/mL), IL-22 (5.74 pg/mL), IP-10 (N/A), MCP-1 (N/A), MIP-1α (macrophage inflammatory protein-1α: 4.53 pg/mL), MIP-1β (macrophage inflammatory protein-1ß: 5.47 pg/mL), PDGF-BB (platelet derived growth factor: N/A), RANTES (regulated on activation, normal T cell expressed and secreted: N/A), TNFα (1.78 pg/mL), VEGF (vascular endothelial growth factor: 7.41 pg/mL).

Blood samples were taken within the first week after onset of fever (≥ 38 °C tympanic). Malaria microscopy and blood cultures were performed as described previously [34]. Blood cultures from the participants of this study remained sterile. A pan-rickettsial PCR targeting the *glt A* gene was performed on samples from patients with negative blood culture (ct-values ranged from 29 to 40). In all of these samples *R. felis* was identified by sequencing of amplicons and BLAST analysis. Details on the molecular methods are described elsewhere [3]. Eight children had a *P. falciparum* co-infection as evidenced by thin and thick blood films.

20 serum samples from age-matched healthy controls from the same geographical area were analyzed in comparison by the LEGENDplex assay. Thin and thick blood films for malaria, as well as PCR examinations for *Plasmodium* sp. and rickettsiae were negative from the control group. Both the *R. felis* infected group and the control group were serologically screened by indirect immunofluorescence tests for IgM and IgG antibodies against *R. felis*. The indirect immunofluorescence tests were performed using *R. felis* (strain California 2) grown in XTC-2 cells. None of the subjects or controls were positive.

Statistical analysis was performed with GraphPad Prism 7 software (GraphPad Software Inc., La Jolla, USA). For comparison between the analyzed groups, 1 way ANOVA and subsequent Tukey’s multiple comparisons test were used. To evaluate the fold change of cytokine concentrations between infected patients and healthy controls, the median of the cytokine concentrations from the mono-infected group and co-infected group, respectively, was divided by the median of the cytokine concentrations of the control group. In addition, ct-values of *R. felis* specific DNA was compared to cytokine concentrations of G-CSF, IL-8, IL-6, IL-10, IP-10, MCP-1, MIP-1α, PDGF-BB and VEGF (Spearman correlation).

## Results

**Fig. 1:**
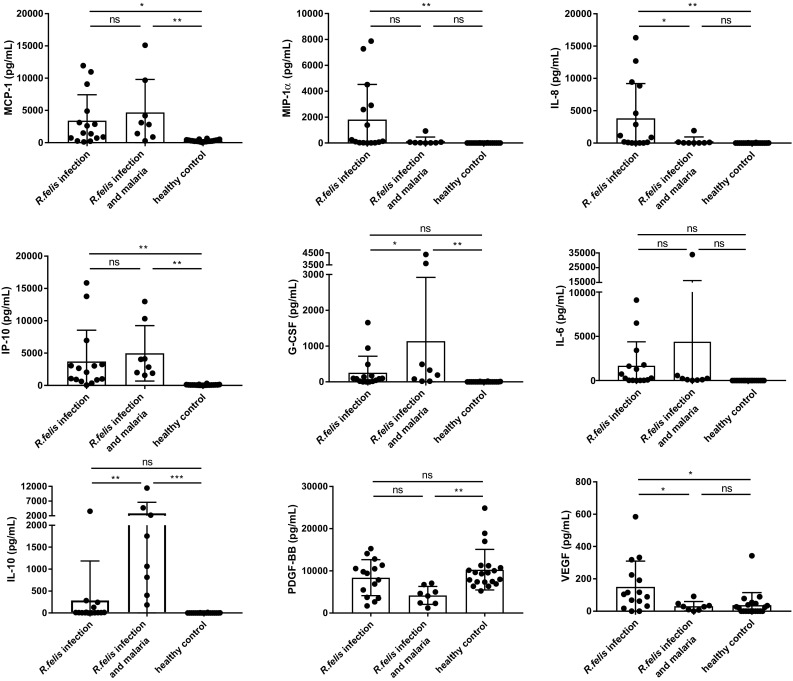
Cytokine and chemokine levels in sera from children during the first week of *R. felis* infection. 15 sera from *R. felis* infected children, 8 sera from *P. falciparum* co-infected children and 20 sera from healthy children without rickettsial disease or malaria were analyzed in parallel by bead-based LEGENDplex assay. Data are expressed as mean ± SD. Statistical analyses was performed with 1 way ANOVA and subsequent Tukey’s multiple comparisons test. Asterisks indicate statistically significant differences (*p < 0.05, **p < 0.01, ***p < 0.001)

Serum levels of IL-8, IP-10, MCP-1, MIP-1α and VEGF were significantly increased in *R. felis* mono-infections in comparison with healthy controls (Fig. 1; Table 1). Of note, the measured cytokine concentrations did not correlate with the detected *R. felis* DNA concentrations. When groups of *R. felis* infected and *P. falciparum* co-infected children were compared, no significant differences were observed in the expression of IP-10, MCP-1 and MIP-1α. In contrast, significantly lower levels of IL-8 and VEGF were found in plasmodial co-infections compared to *R. felis* mono-infections.

**Table 1:** Cytokine changes in infected patients in comparison to healthy controls

Cytokine	Median cytokine concentration (pg/mL) (fold change to healthy control)		
	Healthy control	*R. felis* infected	*R. felis* infected + malaria
IL-6	0	284.6 (NC)	169.0 (NC)
IL-8	6.2	873.6 (141.0)	39.3 (6.3)
IL-10	0	7.6 (NC)	1409.0 (NC)
IP-10	71.6	2042.4 (28.5)	3443.7 (48.1)
G-CSF	0	85.1 (NC)	259.1 (NC)
MCP-1	256.1	1491.8 (5.8)	2949.0 (11.5)
MIP-1α	0	135.5 (NC)	27.5 (NC)
PDGF-BB	9419.8	9418.9 (1.0)	4337.8 (0.5)
VEGF	10.8	104.5 (9.7)	28.4 (2.6)

The median cytokine concentrations and the changes between healthy controls and patients were calculated

*NC* not calculable

IL-6, IL-10 and G-CSF showed smaller, non-significant elevations in *R. felis* mono-infection when compared to healthy controls. The expression of these cytokines was further increased, significantly for G-CSF and IL-10, in co-infected patients compared to both mono-infected patients and healthy controls. Of note, these cytokine concentrations positively correlated with the *P. falciparum* parasitemia [Spearman rank *r*_s_(IL-6) = 0.571, *p* = 0.151; *r*_s_(IL-10) = 0.476, *p* = 0.243; *r*_s_(G-CSF) = 0.619, *p* = 0.115].

PDGF levels were not found to be elevated in mono-infected patients but were significantly reduced in co-infected patients compared to healthy controls.

Serum concentrations of GM-CSF, IL-1ß, IL-2, IL-4, IL-5, IL-9, IL-12p70, IL-13, IL-17A, IL-17F, IL-21, IL-22, IFN-γ, TNF-α, MIP-1ß, RANTES, and bFGF were similar in both patient groups and controls (data not shown).

## Discussion

Only a few studies on cytokines and other inflammatory mediators exist for rickettsial diseases in humans. We hereby report the first data on systemic inflammatory responses in the acute phase of FBSF. Although DNA of *R. felis* has also been found on the skin of healthy individuals [35] and in blood specimens from afebrile persons [36, 37], the negative blood cultures in all *R. felis* infected children examined in our study and the negative blood films for malaria in the specified *R. felis* mono-infected subgroup of our investigation underscore that *R. felis* is the causative agent of illness in our study participants. The fact that *R. felis* plays a role as a pathogen in humans is, also, confirmed by various reports [38–41]. The absence of rickettsial antibodies in the children examined here has been repeatedly reported in patients with PCR-diagnosed *R. felis* infection and is in line with findings of late seroconversion in rickettsial disease in general [40–45].

Levels of IL-8, IP-10, MCP-1, MIP-1α and VEGF were significantly elevated in *R. felis* mono-infection. These chemokines attract immune cells to sites of infection and likely reflect initial host responses to *R. felis*. In line with that, elevations of IL-8 and MIP-1α have been reported in the acute phase of African tick-bite fever (ATBF) and Mediterranean spotted fever (MSF) caused by *R. africae* and *R. coronii*, respectively [46–48]. IL-8, and also IL-6, may play a role in the development of vasculitis resulting from the infection of endothelial cells by mediating the production of acute phase proteins [49]. The levels of IL-6, IL-10 and G-CSF were elevated in mono-infection and increased further in co-infection. Since both IL-6 and IL-10 have been found to be up-regulated in ATBF and MSF as well as IL-10 in malaria, these results suggest common effects of *R. felis* and *P. falciparum* [47, 48, 50, 51]. In contrast, significantly lower levels of IL-8 and VEGF in plasmodial co-infections as compared to *R. felis* mono-infections may indicate opposing effects of *R. felis* and *P. falciparum*. Increased IFN-γ and TNF-α levels, as reported in patients with Japanese spotted fever, ATBF and MSF [6, 47, 48, 52], were not detectable in *R. felis* infected patients. However, this does not exclude a role for IFN-γ or TNF-α in clearing *R. felis* infection at a later point in time.

In our study, immune responses of children towards an infection were determined. Several studies have demonstrated not only diminished humoral responses in children [53] but have also suggested that cell-mediated immunity is not fully developed in children [54]. Furthermore, cytokine production was shown to be reduced in children compared to adults. Keeping this in consideration and the opportunity that children in the median age of 2 years are infected for the first time with these pathogens, immune responses in adults could markedly differ from those of the children. Since children represent a particular vulnerable group, it is even more important to be able to make an early diagnosis to treat them adequately.

The data presented, characterizing cytokine profiles during the first week of infection with *R. felis*, offer new insights in understanding early host immune responses in FBSF and suggest a complex interplay in *R. felis* and *P. falciparum* co-infections. Further studies, such as T cell and antibody analyses are needed to shed more light on the immune responses during *R. felis* infection.

## References

[1] Angelakis E, Mediannikov O, Parola P, Raoult D (2016). *Rickettsia felis*: the complex journey of an emergent human pathogen. Trends Parasitol.

[2] Brown LD, Macaluso KR (2016). *Rickettsia felis*, an emerging flea-borne rickettsiosis. Curr Trop Med Rep.

[3] Sothmann P, Keller C, Krumkamp R, Kreuels B, Aldrich C, Sarpong N (2017). *Rickettsia felis* infection in febrile children, Ghana. Am J Trop Med Hyg.

[4] Richards AL, Jiang J, Omulo S, Dare R, Abdirahman K, Ali A (2010). Human infection with *Rickettsia felis*, Kenya. Emerg Infect Dis.

[5] Socolovschi C, Mediannikov O, Sokhna C, Tall A, Diatta G, Bassene H (2010). *Rickettsia felis*-associated uneruptive fever, Senegal. Emerg Infect Dis.

[6] Sahni SK, Narra HP, Sahni A, Walker DH (2013). Recent molecular insights into rickettsial pathogenesis and immunity. Future Microbiol.

[7] Mansueto P, Vitale G, Di Lorenzo G, Arcoleo F, Mansueto S, Cillari E (2008). Immunology of human rickettsial diseases. J Biol Regul Homeost Agents.

[8] Sahni SK, Rydkina E (2009). Host-cell interactions with pathogenic *Rickettsia* species. Future Microbiol.

[9] Walker DH, Popov VL, Wen J, Feng HM (1994). *Rickettsia conorii* infection of C3H/HeN mice. A model of endothelial-target rickettsiosis. Lab Invest.

[10] Osterloh A, Papp S, Moderzynski K, Kuehl S, Richardt U, Fleischer B (2016). Persisting *Rickettsia typhi* causes fatal central nervous system inflammation. Infect Immun.

[11] Papp S, Moderzynski K, Rauch J, Heine L, Kuehl S, Richardt U (2016). Liver necrosis and lethal systemic inflammation in a murine model of *Rickettsia typhi* infection: role of neutrophils, macrophages and NK cells. PLoS Negl Trop Dis.

[12] Walker DH, Hudnall SD, Szaniawski WK, Feng HM (1999). Monoclonal antibody-based immunohistochemical diagnosis of rickettsialpox: the macrophage is the principal target. Mod Pathol.

[13] Clifton DR, Rydkina E, Huyck H, Pryhuber G, Freeman RS, Silverman DJ (2005). Expression and secretion of chemotactic cytokines IL-8 and MCP-1 by human endothelial cells after *Rickettsia rickettsii* infection: regulation by nuclear transcription factor NF-kappaB. Int J Med Microbiol.

[14] Kaplanski G, Teysseire N, Farnarier C, Kaplanski S, Lissitzky JC, Durand JM (1995). IL-6 and IL-8 production from cultured human endothelial cells stimulated by infection with *Rickettsia conorii* via a cell-associated IL-1 alpha-dependent pathway. J Clin Invest.

[15] Sporn LA, Marder VJ (1996). Interleukin-1 alpha production during *Rickettsia rickettsii* infection of cultured endothelial cells: potential role in autocrine cell stimulation. Infect Immun.

[16] Bechah Y, Capo C, Raoult D, Mege JL (2008). Infection of endothelial cells with virulent *Rickettsia prowazekii* increases the transmigration of leukocytes. J Infect Dis.

[17] Rydkina E, Sahni A, Silverman DJ, Sahni SK (2007). Comparative analysis of host-cell signalling mechanisms activated in response to infection with *Rickettsia conorii* and *Rickettsia typhi*. J Med Microbiol.

[18] Baggiolini M, Clark-Lewis I (1992). Interleukin-8, a chemotactic and inflammatory cytokine. FEBS Lett.

[19] Valbuena G, Walker DH (2009). Infection of the endothelium by members of the order Rickettsiales. Thromb Haemost.

[20] Li A, Dubey S, Varney ML, Dave BJ, Singh RK (2003). IL-8 directly enhanced endothelial cell survival, proliferation, and matrix metalloproteinases production and regulated angiogenesis. J Immunol.

[21] Deshmane SL, Kremlev S, Amini S, Sawaya BE (2009). Monocyte chemoattractant protein-1 (MCP-1): an overview. J Interferon Cytokine Res.

[22] Shi C, Pamer EG (2011). Monocyte recruitment during infection and inflammation. Nat Rev Immunol.

[23] Groom JR, Luster AD (2011). CXCR3 ligands: redundant, collaborative and antagonistic functions. Immunol Cell Biol.

[24] Walker DH, Popov VL, Feng HM (2000). Establishment of a novel endothelial target mouse model of a typhus group rickettsiosis: evidence for critical roles for gamma interferon and CD8 T lymphocytes. Lab Invest.

[25] Li H, Jerrells TR, Spitalny GL, Walker DH (1987). Gamma interferon as a crucial host defense against *Rickettsia conorii* in vivo. Infect Immun.

[26] Walker DH, Olano JP, Feng HM (2001). Critical role of cytotoxic T lymphocytes in immune clearance of rickettsial infection. Infect Immun.

[27] Feng HM, Popov VL, Walker DH (1994). Depletion of gamma interferon and tumor necrosis factor alpha in mice with *Rickettsia conorii*-infected endothelium: impairment of rickettsicidal nitric oxide production resulting in fatal, overwhelming rickettsial disease. Infect Immun.

[28] Moderzynski K, Heine L, Rauch J, Papp S, Kuehl S, Richardt U (2017). Cytotoxic effector functions of T cells are not required for protective immunity against fatal *Rickettsia typhi* infection in a murine model of infection: role of TH1 and TH17 cytokines in protection and pathology. PLoS Negl Trop Dis.

[29] Moderzynski K, Papp S, Rauch J, Heine L, Kuehl S, Richardt U (2016). CD4+ T cells are as protective as CD8+ T cells against *Rickettsia typhi* infection by activating macrophage bactericidal activity. PLoS Negl Trop Dis.

[30] Feng HM, Walker DH (1993). Interferon-gamma and tumor necrosis factor-alpha exert their antirickettsial effect via induction of synthesis of nitric oxide. Am J Pathol.

[31] Turco J, Winkler HH (1984). Effect of mouse lymphokines and cloned mouse interferon-gamma on the interaction of *Rickettsia prowazekii* with mouse macrophage-like RAW264.7 cells. Infect Immun.

[32] Turco J, Winkler HH (1986). Gamma-interferon-induced inhibition of the growth of *Rickettsia prowazekii* in fibroblasts cannot be explained by the degradation of tryptophan or other amino acids. Infect Immun.

[33] Chan ED, Riches DW (1998). Potential role of the JNK/SAPK signal transduction pathway in the induction of iNOS by TNF-alpha. Biochem Biophys Res Commun.

[34] Sothmann P, Krumkamp R, Kreuels B, Sarpong N, Frank C, Ehlkes L (2015). Urbanicity and paediatric bacteraemia in Ghana—a case–control study within a rural-urban transition zone. PLoS One.

[35] Mediannikov O, Socolovschi C, Million M, Sokhna C, Bassene H, Diatta G (2014). Molecular identification of pathogenic bacteria in eschars from acute febrile patients, Senegal. Am J Trop Med Hyg.

[36] Maina AN, Knobel DL, Jiang J, Halliday J, Feikin DR, Cleaveland S (2012). *Rickettsia felis* infection in febrile patients, western Kenya, 2007–2010. Emerg Infect Dis.

[37] Mourembou G, Lekana-Douki JB, Mediannikov O, Nzondo SM, Kouna LC, Essone JC (2015). Possible role of *Rickettsia felis* in acute febrile illness among children in Gabon. Emerg Infect Dis.

[38] Zavala-Castro J, Zavala-Velazquez J, Walker D, Perez-Osorio J, Peniche-Lara G (2009). Severe human infection with *Rickettsia felis* associated with hepatitis in Yucatan, Mexico. Int J Med Microbiol.

[39] Zavala-Velazquez J, Laviada-Molina H, Zavala-Castro J, Perez-Osorio C, Becerra-Carmona G, Ruiz-Sosa JA (2006). *Rickettsia felis*, the agent of an emerging infectious disease: report of a new case in Mexico. Arch Med Res.

[40] Zavala-Velazquez JE, Ruiz-Sosa JA, Sanchez-Elias RA, Becerra-Carmona G, Walker DH (2000). *Rickettsia felis* rickettsiosis in Yucatan. Lancet.

[41] Mediannikov O, Fenollar F, Bassene H, Tall A, Sokhna C, Trape JF (2013). Description of “yaaf”, the vesicular fever caused by acute *Rickettsia felis* infection in Senegal. J Infect.

[42] Mediannikov O, Diatta G, Fenollar F, Sokhna C, Trape JF, Raoult D. Tick-borne rickettsioses, neglected emerging diseases in rural Senegal. PLoS Negl Trop Dis. 2010;4(9) 10.1371/journal.pntd.0000821 PMC2939048 20856858

[43] Zavala-Velazquez JE, Yu XJ, Walker DH (1996). Unrecognized spotted fever group rickettsiosis masquerading as dengue fever in Mexico. Am J Trop Med Hyg.

[44] Fournier PE, Jensenius M, Laferl H, Vene S, Raoult D (2002). Kinetics of antibody responses in *Rickettsia africae* and *Rickettsia conorii* infections. Clin Diagn Lab Immunol.

[45] La Scola B, Raoult D (1997). Laboratory diagnosis of rickettsioses: current approaches to diagnosis of old and new rickettsial diseases. J Clin Microbiol.

[46] Damas JK, Davi G, Jensenius M, Santilli F, Otterdal K, Ueland T (2009). Relative chemokine and adhesion molecule expression in Mediterranean spotted fever and African tick bite fever. J Infect.

[47] Jensenius M, Ueland T, Fournier PE, Brosstad F, Stylianou E, Vene S (2003). Systemic inflammatory responses in African tick-bite fever. J Infect Dis.

[48] Vitale G, Mansueto S, Gambino G, Mocciaro C, Spinelli A, Rini GB (2001). The acute phase response in Sicilian patients with boutonneuse fever admitted to hospitals in Palermo, 1992–1997. J Infect.

[49] Mansueto P, Vitale G, Cascio A, Seidita A, Pepe I, Carroccio A (2012). New insight into immunity and immunopathology of Rickettsial diseases. Clin Dev Immunol.

[50] Noone C, Parkinson M, Dowling DJ, Aldridge A, Kirwan P, Molloy SF (2013). Plasma cytokines, chemokines and cellular immune responses in pre-school Nigerian children infected with *Plasmodium falciparum*. Malar J.

[51] Peyron F, Burdin N, Ringwald P, Vuillez JP, Rousset F, Banchereau J (1994). High levels of circulating IL-10 in human malaria. Clin Exp Immunol.

[52] Cillari E, Milano S, D’Agostino P, Arcoleo F, Stassi G, Galluzzo A (1996). Depression of CD4 T cell subsets and alteration in cytokine profile in boutonneuse fever. J Infect Dis.

[53] Rijkers GT, Sanders LA, Zegers BJ (1993). Anti-capsular polysaccharide antibody deficiency states. Immunodeficiency.

[54] Lilic D, Cant AJ, Abinun M, Calvert JE, Spickett GP (1997). Cytokine production differs in children and adults. Pediatr Res.

